# Altaids, continental growth and metallogeny

**DOI:** 10.1093/nsr/nwad004

**Published:** 2023-01-09

**Authors:** Wenjiao Xiao

**Affiliations:** Xinjiang Institute of Ecology and Geography, Chinese Academy of Sciences, China

The Altaids, occupying vast tracts between the Baltica and Siberia Cratons to the north and the Tarim and North China Cratons to the south, was constructed by multiple subductions of the Paleo-Asian Ocean (PAO) from the Neoproterozoic to early Mesozoic [1,2]. The Altaids typifies the largest accretionary orogenic collage on Earth and holds key information regarding cutting-edge theories of orogenic architecture and continental growth and economic benefits [2]. This Special Topic in 2023 collects two Research Highlights, three Perspectives and three Reviews addressing the central issues of the accretionary orogenesis, continental growth and metallogeny of the enormous Altaids.

The Altaids is composed of three major collages (the Mongolia collage in the north, the Kazakhstan collage in the west, and the Tarim–North China collage in the south) [2]. Soldner [3] presents a review of age, temperature, pressure and thermobaric ratio of the metamorphic rocks in the Tarim–North China collage and highlights two distinct but successive orogenic cycles, corresponding to peripheral-accretionary and interior-collisional orogenic phases, respectively. Schulmann *et al*. [4] combine newly acquired paleomagnetic, structural and geochronological data from the Mongolia collage to demonstrate that the counterclockwise rotation of its southern limb was strongly linked with polyphase deformation in the Mongolian Altai Wedge and Trans-Altai Zone, three-stage subduction-collisional evolution of the Solonker suture, and the final closure of the PAO and Mongol-Okhotsk Ocean. Liu *et al*. [5] summarize long-lived subduction-accretion processes of the three major Kazakhstan, Mongolia and NE China oroclines and reveal their two-stage curvature mechanisms.

The Altaids encompasses the largest and most productive juvenile crustal growth for the Phanerozoic, particularly in some special tectonic positions where most productivity of juvenile material was best preserved [6,7]. Through detailed geological mapping in the Beishan, Hong *et al*. [8] uncover the distinct Andean-type continental arc and oceanic arc-back-arc systems, as well as multi-phase arc accretion/collision in the Permian during the closure of the PAO. Safonova and Perfilova [9] reconstruct the survived and disappeared fossil intra-oceanic arcs in central and eastern Kazakhstan to manifest that accretionary growth and subduction erosion alternatively functioned at the active margins of the PAO in Paleozoic time. Using the powerful method of comparative orotomy, Kusky and Şengör [10] compare and contrast the comprehensive geological materials and mechanisms of the Archean Superior and Phanerozoic Altaid orogenic systems, revealing remarkable similarities in accretionary orogenic processes, spatial and temporal scales of structural progression, and formation of continents on Earth over time. Applying isotopic contour mapping, Wang *et al*. [11] highlight the three-dimensional lithospheric compositional architecture of the Altaids and further reveal the control relationship of the juvenile crustal growth to crustal reworking on the specific ore resources in the region. Aitchison [12] retraces the research heritage of the Altaids since the eighteenth century and identifies the methodological issues that arose in modern investigations. In addition to focusing on consumption of ancient ocean basins and concomitant orogenesis, the opening of early oceanic spaces and the location of former continent-ocean boundaries with relics of ocean-continent transitions and/or oceanic metamorphic complexes remain to be tested and resolved.
